# Dragées of Iron, Manna, and Bismuth

**Published:** 1860-04

**Authors:** 


*Dragées of Iron, Manna, and Bismuth.* By Dr. Morin.

The objections to ferruginous preparations may be thus summed up: if they are insoluble, they are frequently useless; if soluble, repugnant to the taste, and fatiguing to the stomach and intestines. The new preparation suggested by Foucher, a pharmacien of Orleans, combines all the advantages claimed for preparations of iron, while it presents none of the inconveniences for which they have so justly been reproached. The formula is as follows:

| | |
|---|---|
| ℞.—Ferri pyrophosphat., | 0 05 gramme. |
| Bismuthi subnitrat., | 0 05 " |
| Mannæ puriss., | 0.25 " |

This is sufficient for one dragée. These medicinal agents, being thoroughly mixed, will form dragées easily chewed. They are called *manna-bismuthic dragées of iron.*

The iron salt, the pyrophosphate, is a fixed salt, constant in its composition, perfectly soluble, without injurious action on the stomach, not decomposable by the acid products of that viscus, and most easily absorbed and assimilated. So much for the true active principle of these dragées. But it is useless to dwell upon *this*, since academic reports, as well as clinical experience, demonstrate the advantages of this salt. As for the subnitrate of bismuth, practice has, to a certain extent, indicated that its addition to iron is indicated logically. Hence, for a long time, a prominent physician of Paris, well known for his protracted investigations on the physiology and pathology of the digestive organs, Dr. Gaubert, has used the subnitrate of bismuth in all young, lymphatic subjects, possessed of great nervous susceptibility of the intestines, whose health demanded the employment of tonics. Subnitrate of bismuth is the hackneyed remedy, so to speak, for all gastralgic pains. Its addition is so physiological, so rational, that it might be asked how it happened that its application, as made by Foucher, had not been made by every one before.

The manna has a double effect: it combats the constipation which the ferruginous preparations excite, and at the same time maintains the pyrophosphate in a permanent state of solution—thus allowing of its absorption throughout the whole extent of the digestive apparatus.

A word must be said as to the form of this preparation. It is almost a case where one might employ Cuvier's expression, " The form is more essential than the substance !" To say *more* essential would be going too far; we shall content ourselves by saying that a medicament in the form of a dragée—a true *bonbon*—particularly pleasant for children, is something not to be despised in numerous diseases, where martial preparations are needed.

To conclude, if we may be allowed to institute a comparison between Foucher's preparation and the other ferruginous preparations, we would say that his dragées are agreeable to the taste, whilst most of the others are disagreeable. His is inoffensive to the stomach, whilst most of the others fatigue and irritate that organ; his does not produce constipation, the others provoke a tedious obstruction of the digestive organs; his remains soluble in the midst of the digestive mucosities, and is promptly and completely assimilated; the others pass through the bowels without being assimilated.—*Gazette des Hôpitaux.*

